# Monitoring the Spatial Distribution of Cover Crops and Tillage Practices Using Machine Learning and Environmental Drivers across Eastern South Dakota

**DOI:** 10.1007/s00267-024-02021-0

**Published:** 2024-07-29

**Authors:** Khushboo Jain, Ranjeet John, Nathan Torbick, Venkatesh Kolluru, Sakshi Saraf, Abhinav Chandel, Geoffrey M. Henebry, Meghann Jarchow

**Affiliations:** 1 Department of Sustainability and Environment, University of South Dakota, Vermillion, SD 57069 USA; 2 Department of Biology, University of South Dakota, Vermillion, SD 57069 USA; 3 Agreena, Langebrogade 3F, 3rd Floor, 1411 Copenhagen, Denmark; 4 Department of Geography, Environment, and Spatial Sciences, and Center for Global Change and Earth Observations, Michigan State University, East Lansing, MI 48824 USA; 5 Center for Global Change and Earth Observations, Michigan State University, East Lansing, MI 48823 USA

**Keywords:** Conservation tillage, Cover crops, Sentinel-2, Environmental variables

## Abstract

The adoption of conservation agriculture methods, such as conservation tillage and cover cropping, is a viable alternative to conventional farming practices for improving soil health and reducing soil carbon losses. Despite their significance in mitigating climate change, there are very few studies that have assessed the overall spatial distribution of cover crops and tillage practices based on the farm’s pedoclimatic and topographic characteristics. Hence, the primary objective of this study was to use multiple satellite-derived indices and environmental drivers to infer the level of tillage intensity and identify the presence of cover crops in eastern South Dakota (SD). We used a machine learning classifier trained with in situ field samples and environmental drivers acquired from different remote sensing datasets for 2022 and 2023 to map the conservation agriculture practices. Our classification accuracies (>80%) indicate that the employed satellite spectral indices and environmental variables could successfully detect the presence of cover crops and the tillage intensity in the study region. Our analysis revealed that 4% of the corn (*Zea mays*) and soybean (*Glycine max*) fields in eastern SD had a cover crop during either the fall of 2022 or the spring of 2023. We also found that environmental factors, specifically seasonal precipitation, growing degree days, and surface texture, significantly impacted the use of conservation practices. The methods developed through this research may provide a viable means for tracking and documenting farmers’ agricultural management techniques. Our study contributes to developing a measurement, reporting, and verification (MRV) solution that could help used to monitor various climate-smart agricultural practices.

## Introduction

The Euro-American colonization of the U.S. Midwest in the 19th century transformed 100 million hectares of prairies into predominantly annual row-crop farming systems (Yu et al. 2020). This shift led to extensive soil carbon losses, which have increased atmospheric greenhouse gas concentrations and reduced soil fertility (Bernacchi et al. 2005; Lal 2019). In response, climate-smart agriculture has been promoted by both government and industry to ensure sustainable food production and combat these losses (Bai et al. 2019). Recognizing the potential for sequestering more carbon in the soil, combined with incentive programs, more farmers have started adopting practices such as conservation tillage and cover cropping to reduce soil disturbance and increase soil carbon inputs (Guto et al. 2012; Deines et al. 2019; Zheng et al. 2013).

Conservation tillage practices, including minimum and no-tillage, are effective in minimizing soil disturbance, preserving soil fertility, and maintaining a crop residue cover of greater than 15% (Claassen et al. 2018; Karlen et al. 1994; Smith et al. 2010; Sprunger et al. 2021). Often minimum tillage schema leaves a crop residue cover between 15 and 30%, while no-tillage can leave crop residue of more than 30% (Gustafson et al. 2019). In contrast, conventional agricultural techniques, such as plowing or conventional tillage, typically leave less than 15% of the crop residue on the soil surface (Claassen et al. 2018). These conventional practices increase soil disturbance and accelerate the loss of soil organic matter by elevating oxygen levels within the soil profile. This process increases the microbial conversion of organic carbon, resulting in low soil aggregate stability (Cooper et al. 2021). As of 2023, the adoption rate of no-till farming in the U.S. Midwest remains relatively low, at approximately 40% (Kwang et al. 2023). Previous studies have shown that under current conventional tilling practices, the U.S. Midwest faces an estimated loss of about 9.7 billion tons of soil over the next century, potentially leading to substantial economic losses (Kwang et al. 2023; Thaler et al. 2022).

In addition to conservation tillage, other practices like cover cropping can significantly improve soil health by increasing the quantity of roots and root exudates in the soil. Cover crops, planted between two primary crops, are essential for maintaining productivity and ensuring adequate soil coverage during the fallow period (Wittwer and van der Heijden 2020). Lichtenberg (2004) observed that economic variables, such as enhanced profitability resulting from higher crop production and additional cash generated from haying and grazing, significantly impact the adoption of conservation practices. Despite their benefits, cover crops adoption remains relatively low at 7.2% in U.S. Midwest (Zhou et al. 2022). The limited expansion of cover cropping and conservation tillage can be attributed to challenges such as the need for specialized planting equipment, increased farm management activities, and labor constraints.

**Fig. 1:**
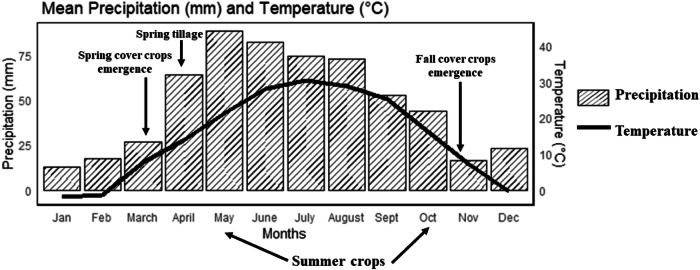
Mean monthly precipitation (mm) and temperature (°C) changes in eastern South Dakota from 2011 to 2021, with the general timing of cover crop and tillage establishment practices

Additionally, the U.S. Midwest experiences hot and humid summers along with cold and snowy winters, which limit plant growth and contribute to winter kill of plants (Janowiak et al. 2016) (Fig. 1). Furthermore, the adoption of no-tillage and cover cropping, along with their associated benefits varies based on soil characteristics, crop rotation, and climatic regimes. Previous studies showed that cover crop adoption is more prevalent in regions with low soil organic matter and high erodibility (Bowman and Wallander, 2021). While cover crops can facilitate successful planting by absorbing excessive soil moisture in mesic regions, they may reduce the amount of water available to the cash crop in arid and semiarid areas (Reese et al. 2014; Wang et al. 2021). Cover-crop benefits may also be limited by the shorter growing season, harsh winters affecting plant survival, and variable fall and spring weather in northern climates (Reese et al. 2014).

Previous research also indicates that conservation tillage which leaves high residue cover can potentially impede the timely germination of plants and cause a decline in crop yield (Blanco-Canqui and Lal 2009; Nouri et al. 2021, 2019). Conversely, in drier regions of the U.S. Midwest, the presence of crop residue has been to shown to help increase crop water availability and yield (Blanco-Canqui and Lal 2009). This spatial variability in the benefits of conservation cover crop and tillage use necessitates developing models over large spatial scales using remote sensing, especially in areas with short growing seasons and water stress (Farmaha et al. 2022; Reese et al. 2014).

Remote sensing advancements in analytical capabilities (e.g., cloud computing, machine learning, data fusion) provide an opportunity to monitor cropping systems at a large spatial scale and over extended periods using multispectral and synthetic aperture radar (SAR) imagery (Deines et al. 2019). Recent studies have used both airborne (Wang et al. 2023; Yuan et al. 2019) and spaceborne (Azzari et al. 2019; Seifert et al. 2018) sensors to quantify cover crop growth and tillage intensity.

We used these advancements in satellite data sensors and modeling systems for a field-scale assessment of conservation agriculture aimed at supporting the Measurement, Reporting, and Verification (MRV) of climate-smart practices. Our study aims to address the following challenges highlighted in previous research to examine the spatial distribution of cover cropping and tillage practices. Previous research showed that crop residue and soil typically exhibit distinct spectral signatures only at particular wavelengths (Nagler et al. 2003; Gao et al. 2022). Therefore, we seek to employ few bands that are located at wavelengths of 1600 and 2300 nm which can help detect cellulose, lignin, and hemicellulose in crop residues (Nagler et al. 2003; Zheng et al. 2014). These wavelengths can also help distinguish the spectrum reflectance features of green vegetation, soil, and crop senescence (Cai et al. 2019).

In addition, optical bands fail to provide clear images during cloudy days (Jennewein et al. 2022). Therefore, we seek to employ SAR imagery, which operates in the microwave spectrum, and offers an additional data source capable of collecting imagery during nighttime and cloudy days (McNairn et al. 1998). Finally, monitoring variations in soil surface roughness, moisture content, and alterations in the structure of crop residue resulting from tillage activities poses a significant challenge (Jennewein et al. 2022; Zheng et al. 2014). Studies have shown that each tillage instrument applies unique pressure, compaction, and disturbance patterns to the soil, affecting surface roughness and structure (Najafi et al. 2018a, 2018b). To address this we seek to implement gray-level co-occurrence matrix (GLCM) texture variables which has the considerable advantage of capturing variations in soil texture and structure induced by tillage (Azzari et al. 2019; Najafi et al. 2018a; Zhao et al. 2016).

Our study is built to help address gaps in the application readiness levels of cover crop and tillage mapping with an application to support climate smart monitoring in the Northwestern Corn Belt. We focus on eastern South Dakota (SD), a region in the Northwestern Corn Belt characterized by a diverse combination of grasslands and cropland (O’Brien et al. 2020). The study area is also characterized by a climate transitional zone that shows a decrease in precipitation from east to west (Wang et al. 2021). Therefore, we used various environmental drivers and variables derived from satellite sensors to monitor the growth of cover crops and tillage intensity.

The overarching research questions include: (1) What is the spatial distribution of cover crops at field scale and tillage practices across eastern SD? (2) What climatic, soil, and topographic factors correlate with the adoption of on-farm conservation practices? The objectives of this study are threefold: (i) to develop a machine learning approach to map the extent of cover crops at the field scale, (ii) to map tillage practices at the field scale, and (iii) to evaluate the relative importance of site-specific conditions on the adoption of different conservation agriculture practices. These outcomes will help track climate-smart adoption and farm-level factors driving decision making.

## Materials and Methods

### Study Area

**Fig. 2:**
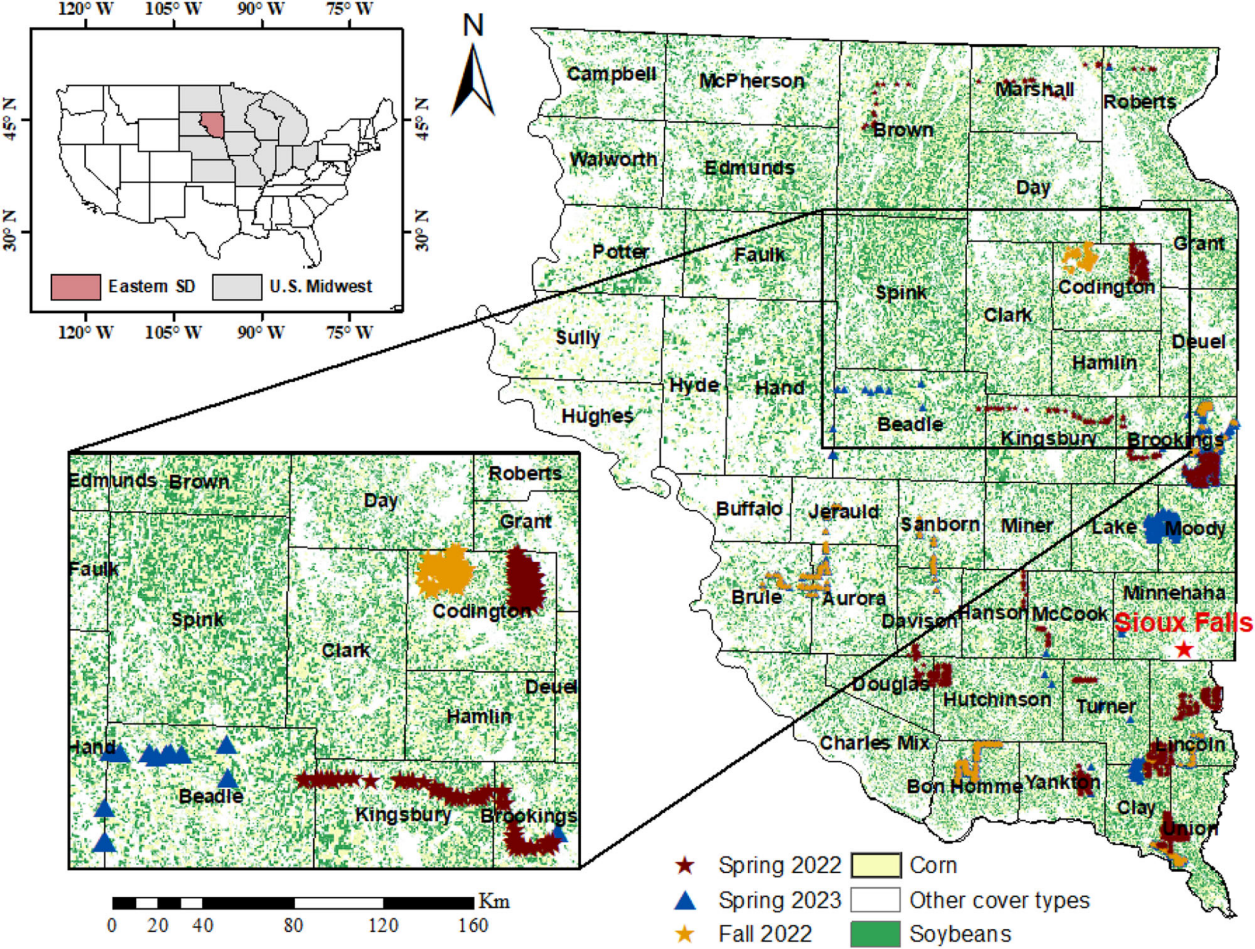
Locations of field data collections (*n* = 1394) for the years 2022–2023, overlaid on the crop data layer (2022) with county boundaries

Agriculture is the most prominent industry in South Dakota, with approximately 75% (>40,000 km^2^) of its farmed land dedicated to the cultivation of corn (*Zea mays*) and soybeans (*Glycine max*) (Kolady et al. 2021) (Fig. 2). The 100th meridian and the Missouri River pass roughly through the center of South Dakota, dividing it into roughly equal regions. The eastern half has productive farmland with adequate rainfall for row crops, whereas the western half of the state is semiarid (Kolady et al. 2021; Bishop et al. 2021). The yield, spatial distribution, and type of cropland and natural vegetation are particularly vulnerable to soil moisture and drought variability. As a result, rain-dependent agricultural regions in the eastern SD may be forced to contend with significantly altered extreme weather patterns.

This region has a continental climate, with corn and soybean fields planted in late April-May and harvested in September-October (Pikul et al. 2008). Farmers typically harvest the grain from most corn and soybean fields once the plants have reached senescence. Sometimes, farmers harvest corn early for silage, giving more time for cover crops to grow before winter (Nowak et al. 2021). In 2022, SD harvested around 6.6 million tons of green corn to produce corn silage (USDA-NASS 2023). This phenomenon is particularly prevalent in regions in the vicinity of dairy operations, cattle feedlots, or low-grain years due to drought (Katsvairo and Cox 2000).

### Field Data

We conducted field-edge surveys in 1394 crop fields across eastern SD, which involved visual observations made from the perimeter of the fields. We used the collected data from these surveys to train, test, and validate our models. Cover crop and tillage data were gathered in April/May of 2022 (*n* = 894) and 2023 (*n* = 500) to document field conditions following winter snow melt and spring cover crop development, respectively, before the substantial growth of corn and soybeans (Fig. 2). Additionally, we collected field data in November 2022 (*n* = 112) to record conditions in the fields after the senescence of cash crops, fall tillage, and the emergence of fall cover crops. At each field, we recorded the approximate amount of crop residue cover, presence of living plants, previous crops, tillage equipment employed, intensity of soil disturbance, and type of tillage (conventional, minimum, or no-tillage). We visited some of the fields twice, in the fall and spring, to validate the timing of tillage, the harvest of previous crops, and the establishment of new crops. We mainly identified winter wheat (*Triticum aestivum*), rye (*Secale cereale*), oats (*Avena sativa*) and alfalfa (*Medicago sativa*) species planted as cover crops on fields. We also captured vertical and oblique photographs to document the cover crop and tillage conditions for each location.

### Data Sources

We used Google Earth Engine (GEE) to obtain dual-polarization C-band SAR Sentinel-1 VV and VH bands processed to backscatter coefficient (σ°) in decibels (dB). Additionally, we incorporated Sentinel-2 Level 2A Bottom-Of-Atmosphere (BOA) corrected reflectance products, which include bands 2–8, 11, and 12. We applied cloud and cloud shadow masks to ensure data accuracy using the quality assessment (QA) band on the satellite images. Images for tillage detection were acquired in spring, from May 1 to May 30, for 2022 and 2023.

We used GEE to incorporate ten surface texture metrics from each band’s gray level co-occurrence matrix (GLCM). These metrics capture nuanced textural characteristics across different features within the images, enhancing our ability to represent spatial variations effectively. This approach can be beneficial in improving the accuracy of crop residue detection on the ground (Azzari et al. 2019; Xiang et al. 2022). For the cover crop model, we used Sentinel-2 Level 2A images from late October to early December in 2022 to limit the inclusion of unharvested summer crops, such as corn and soybeans, which can persist late into the year. Additionally, we used mid-March to late-April images to identify areas where cover crops were present during the spring months. Sentinel-2 level 2A bands were used to calculate the six vegetation indices: normalized difference vegetation index (NDVI), normalized difference tillage index (NDTI), normalized difference index 5 (NDI5), normalized difference index 7 (NDI7), normalized difference residue index (NDRI), and simple normalized difference vegetation index (SNDVI). These indices were instrumental in characterizing vegetation and land cover dynamics across the study area, as detailed in Supplementary Tables 1 and 2.

The cropland data layer (CDL) produced at 30 m resolution by the United States Department of Agriculture (USDA) National Agricultural Statistics Service (NASS) was used to obtain cropping pattern trends (USDA-NASS 2024). We obtained daily precipitation and temperature data at 4.6 km spatial resolution from the Prism dataset (https://prism.oregonstate.edu/) (Rupp et al. 2022). From this data, we computed seasonal cumulative precipitation for both fall (mid-October to mid-December) and spring (mid-March to mid-May) in 2022 and 2023. Accumulated growing degree days (AGDD) were calculated using the daily average temperatures (T_mean_) during the cover crop growing season (October to April)(Kc et al. 2021). The base temperature (T_base_) was set at 0 °C, since it is suitable for many cover crop varieties (Mcmaster and Wilhelm 1997).

We also acquired daily land surface temperature (LST) data at 1 km resolution from the Moderate Resolution Imaging Spectroradiometer (MODIS) MOD11A1 V6.1 dataset to characterize surface temperature changes due to cover crop and tillage growth. To generate elevation and slope, we used SRTMGL1, a NASA Shuttle Radar Topography Mission Global 1 arc-second (~30 m) V003 product (NASA 2020; Wan et al. 2015). Soil textural information was obtained from the Soil Survey Geographic (SSURGO) database of the USDA (NRCS 2016). We acquired daily surface soil moisture values at 0–5 cm depth from the Soil Moisture Active Passive (SMAP) L-Band dataset at 9 km spatial resolution (Chaubell et al. 2020). We procured black sky albedo data from the MODIS MCD43A3 V6.1 dataset, which has a resolution of 500 m. This information is crucial because the presence of cover crops and residue cover on a farm reduces the amount of solar energy absorbed by the surface, potentially cooling the surrounding region (Davin et al. 2014). Finally, we utilized the University of Idaho’s Gridded Surface Meteorological Dataset (gridMET) and TerraClimate to determine the vapor pressure deficit (VPD) and wind speed for each pixel in the study area at 4 km resolution (Abatzoglou 2013). The derived bands and layers were resampled to a resolution of 10 m using the standard resampling methods available in ArcGIS 10.2, to ensure consistency and compatibility across datasets (Xiang et al. 2022) (Supplementary Table 3).

The crop field boundaries were originally delineated using the 2022 and 2023 Cropland Data Layer (CDL) layers, and then rectified using high-resolution images from Google Earth Pro (Azzari et al. 2019; Luo et al. 2023). Field boundaries were buffered inward by 20 m, to mitigate georectification errors and exclude impacts from fencerows and buffer strips (Gelder et al. 2009). Fields smaller than 100 m² were excluded from the analysis to minimize adjacent effects from non-crop land classes, such as forests, roads, and grass waterways (Zhou et al. 2022). For the tillage model, we randomly sampled five pixels from each field, ensuring a minimum distance of 1 meter between each sample, to capture spatial variability and enhance model performance (Azzari et al. 2019). Conversely, in the cover crop model, we opted not to increase the pixel sampling size due to the binary nature of our field dataset (presence or absence of cover crops) and the observed uniformity in pixel values within vegetated fields.

### Machine Learning Modeling

We developed two models using the collected field data: one to predict the growth of cover crops and another to determine the tillage intensity. We specified three tillage classes for our tillage model: conventional, minimum, and no-tillage farming practices. For our cover crop model, we have identified three distinct categories: (i) Winter Kill - representing cover crops planted in the fall, grew during the fall season, but were undetected the subsequent spring; (ii) Winter Hardy - signifying cover crops planted in the fall that were established during the fall season and grew the following spring; and (iii) Spring Emergent - indicating cover crops not detected in the fall but emerged during the following spring season.

**Fig. 3:**
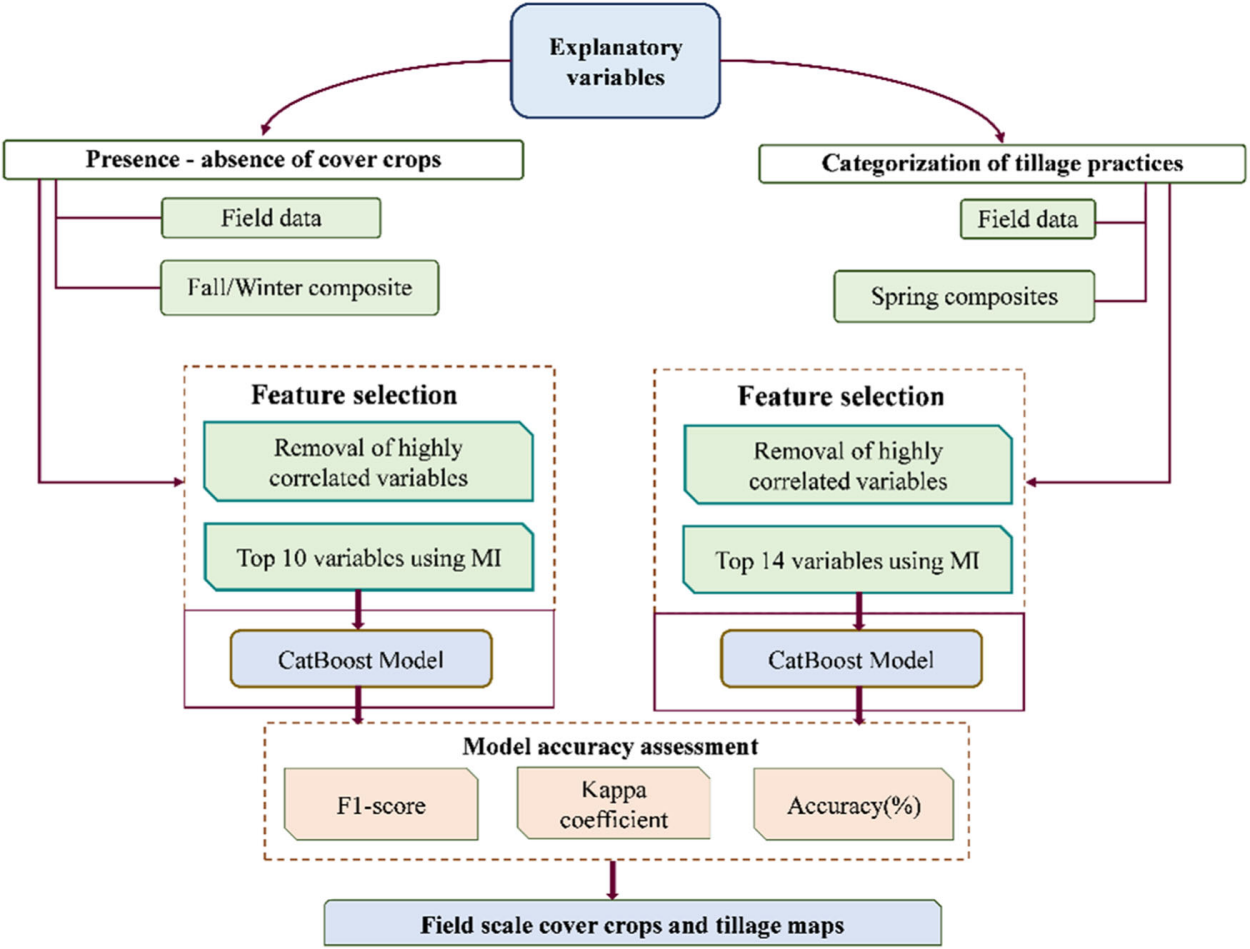
Schematic workflow for classifying tillage types and cover crops distribution across eastern South Dakota

We used a machine-learning classification approach, to develop models for detecting cover crop presence and tillage intensity at a pixel level across eastern SD (Fig. 3). We used the CatBoost package, a powerful gradient boosting library developed specifically for handling categorical features, in the Python programming language (Ibrahim et al. 2020; Prokhorenkova et al. 2018). To enhance model accuracy and efficiency, we applied feature selection techniques. Initially, a Pearson correlation threshold (r > 0.8) was employed to remove highly correlated variables, ensuring model stability and reducing redundancy (Taylor and Bates 2013; Venkatesh et al. 2022; Zheng et al. 2012). Additionally, we used mutual information, a method that evaluates the level of independence between each predictor and the response variable, effectively accommodating nonlinear relationships (Herrera et al. 2015; Li et al. 2016). The model was re-iterated to eliminate all the predictors with the lowest significance scores, resulting in an optimized subset of predictors that are detailed in Tables 1 and 2.

**Table 1:** List of independent variables selected for the cover crop model in decreasing order of their importance

S. no	Independent variables	Codes
1.	Normalized Difference Vegetation Index	NDVI
2.	Normalized Difference Tillage Index	NDTI
3.	Precipitation (fall/spring)	PPTc
4.	Surface texture	texture
5.	Wind velocity (fall/spring)	Wind_speed_C_
6.	Growing degree days (fall/spring)	GDD
7.	Land surface temperature (fall/spring)	LSTc
8.	Surface soil moisture (fall/spring)	SSM_C_
9.	Slope	slope
10.	Sentinel-1 SAR VH	VH

Subscript C indicates that the variables were used for the cover crop model

**Table 2:** List of independent variables selected for the tillage model in decreasing order of their importance

	Independent variables	Codes
1.	Spring land surface temperature	LST_T_
2.	Spring precipitation	PPT_T_
3.	Spring wind velocity	Wind_speed_T_
4.	Spring surface soil moisture	SSM_T_
5.	Spring albedo	Albedo_T_
6.	Surface texture	texture
7.	Sentinel 2 Level 2A-Shortwave InfraRed 1	B11
8.	Normalized Difference Tillage Index	NDTI
9.	Sentinel 2 Level 2A-Near InfraRed	B8
10.	Normalized Difference Vegetation Index	NDVI
11.	Normalized Difference Residue Index	NDRI
12.	Crop Residue Cover Index	CRC
13.	Normalized Difference Index 5	NDI5
14.	Slope	slope

Subscript T indicates that the variables were used for the tillage model

Subsequently, we trained the machine learning algorithms using this optimized predictor set. Pixel-level predictions were aggregated to the field scale using ArcGIS’s zonal statistics tool, facilitating the classification of fields based on the majority of specific pixel types detected. Notably, only pixels classified as corn and soybeans from 2021 and 2022 CDL datasets from USDA-NASS were utilized for training the model, ensuring relevance and accuracy in agricultural land-use classification (USDA-NASS 2024).

For both models we used the bootstrapping method (including replacement) to divide the samples into 80% for training and 20% for testing the cover crop and tillage detection models (Saraf et al. 2023). Azzari et al. (2019) suggested that the pixels within each field are likely to be highly correlated and should not be split into training and validation subsamples, as that would result in overestimating the model performance. Therefore, the training dataset consisted of pixels extracted from 80% of the fields, whereas the remaining 20% were used to test the classification model (Seifert et al. 2018).

We implemented hyperparameter tuning to optimize performance of our classification task (Prokhorenkova et al. 2018). We computed class weights using Scikit-learns ‘compute_class_weight’ function, to address class imbalance ensuring each class was appropriately weighted during training (Pedregosa et al. 2011). For hyperparameter tuning, we defined a grid of potential values for key hyperparameters such as iterations, depth, learning rate, and border count. Using Grid Search with cross-validation, we identified the best combination of hyperparameters. The L2 regularization for leaf weights explored values 1, 3, 5, and 10, which helped to control the complexity of the model and prevent overfitting, while the border count values were set at 128. The final model was configured with 500 boosting iterations and set to the optimal values found during tuning (Najafi et al. 2018a, 2018b). The evaluation metric was set to ‘MultiClass’, and verbose was set to 100 for regular updates. By systematically tuning these hyperparameters, we optimized our CatBoost model for improved classification performance (Pedregosa et al. 2011).

We used the testing dataset to assess the accuracy of the trained model. We generated a confusion matrix to evaluate the accuracy of the classifier, which illustrated the distribution of the classification results for the testing data concerning their actual classes. Furthermore, we calculated various metrics, including overall accuracy, kappa coefficient, and F1-score, to evaluate the classifier’s effectiveness.

## Results

The relative accuracies of the cover crop and tillage models were evaluated to better understand the predictive capability of environmental, topographical, and Sentinel-based indices within a cropland setting. The main output of the CatBoost models developed in this research consist of field-scale maps of cover crops and tillage practices with a spatial resolution of 10 m.

### Cover Crop Model

The model built to determine the presence or absence of cover crops had an out-of-sample accuracy of 85%. Given the binary nature of the data, more appropriate performance measures, such as the kappa coefficient, F1-score, and accuracy, registered metrics of 0.70, 0.94, and 0.84 (presence of cover crops), respectively (Table 3). We utilized the Pearson correlation method with a threshold of <|0.8| to verify the absence of correlations among our predictor variables (Taylor and Bates 2013) (Supplementary Fig. 1). The confusion matrix depicted high accuracy for predicting both the presence (40%) and absence (45.19%) of cover crops (Supplementary Table 4). Subsequently, we identified ten predictor variables that exhibited the highest mutual information scores, indicating their potential to explain the greatest degree of spatial variability in cover crop presence and absence (Table 2). Variable importance ranked NDVI as the most important variable based on the CatBoost model feature importance scores for cover crop mapping, followed by NDTI, land surface temperature, wind speed, growing degree days, and surface texture.

**Table 3:** Classification report for tillage and cover crop models

Model	Precision	Recall	F1-score	Metrics
Tillage				
Conventional tillage	0.78	0.71	0.74	
Minimum tillage	0.83	0.90	0.86	
No-tillage	0.88	0.78	0.83	
Accuracy				0.83
Kappa				0.72
Cover crops				
Absence	0.81	0.89	0.84	
Presence	0.90	0.82	0.86	
Accuracy				0.85
Kappa				0.70

Our findings indicated that areas with more accumulated growing degree days (>150 days) during the fall months were more associated with cover cropping. Similarly, areas experiencing rainfall exceeding 80 mm in the fall exhibited a greater concentration of cover cropping. In general, our study determined that seasonal NDVI, which measures the amount of vegetation cover between the fall and spring, as well as land surface temperature (LST_C_), growing degree days (GDD), and precipitation (PPT_C_), which influence crop performance during the growing season, were the most important factors in forecasting cover crop practices. These variables significantly influence crop performance during the growing season, making them essential for mapping cover crop adoption.

**Fig. 4:**
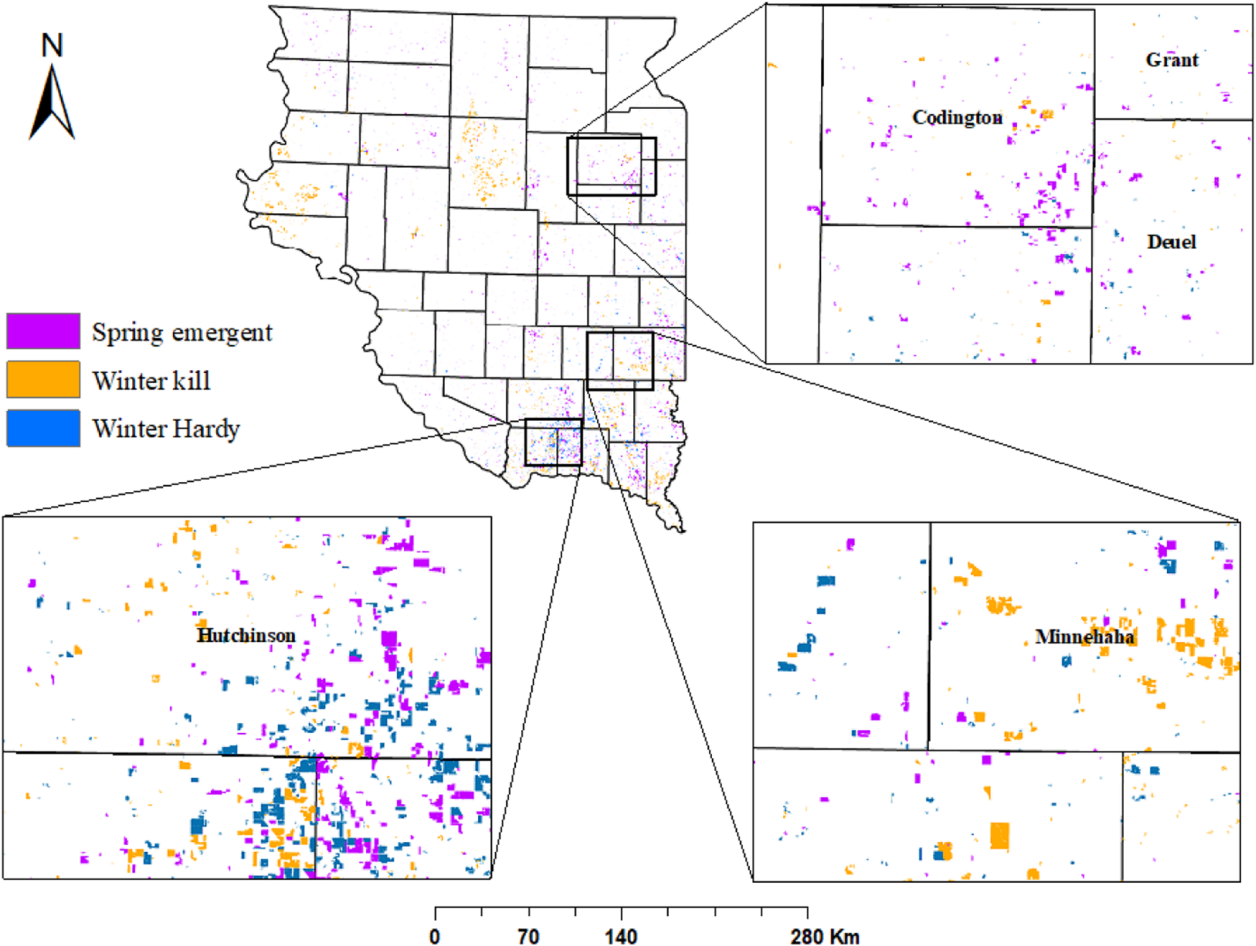
Field-level classification of cover crop plantings in fall 2022 and spring 2023

Our cover crop model estimated that 4% (1561 km^2^) of the corn-soybean fields in eastern SD had a cover crop growing in either fall 2022 or spring 2023 (Fig. 4). Our results indicated that approximately 590 km^2^ of fields with cover crops were spring emergent, 664 km^2^ of cover-cropped fields experienced winter kill, and 307 km^2^ of fields with cover crops were classified as winter hardy. We found a high frequency of cover cropped fields in the southeastern regions of eastern SD especially Hutchinson, Bon Homme, Yankton, Codington, and Minnehaha counties. In contrast, cover crops were generally not planted in the western part of eastern SD.

Cover cropping was also more prevalent in eastern counties such as Minnehaha, Turner, Brookings, and Codington, where silt content was greater than 58% and clay concentration was greater than 18% (Supplementary Fig. 2). The results indicate that cover cropping in fall, particularly in fields previously used for corn cultivation is more widespread especially in the Bon Homme County. Specifically, 25% of corn and soybean fields in Bon Homme County had cover crops. Additionally, throughout eastern SD, nearly 80% of the cover crops were planted after the harvest of corn, as opposed to soybeans.

### Tillage Model

**Fig. 5:**
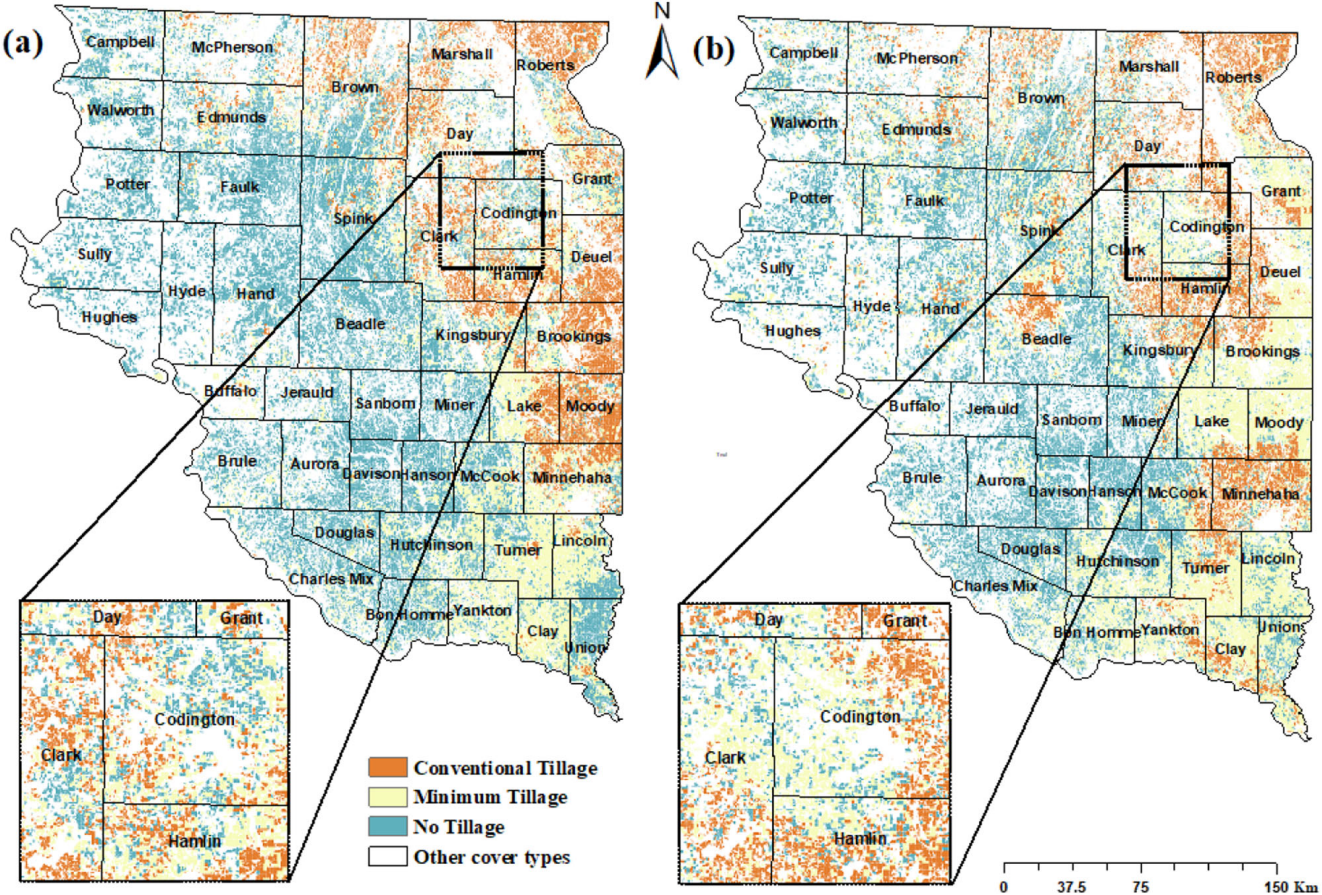
Field-level classification of tillage types for (**a**) 2022 (left) and (**b**) 2023 (right) across eastern SD using the CatBoost classifier

For our tillage model, we selected 49 out of 137 predictor variables based on the correlation (r) threshold of <|0.8| (Supplementary Fig. 3). We calculated mutual information and selected the 14 predictor variables that could explain the most spatial variability of tillage types (Figs. 4 and 5). Land surface temperature (LST), precipitation, wind speed, soil moisture, and albedo were the most important predictor variables in the tillage model. The CatBoost model demonstrated an accuracy of 0.82 and a kappa coefficient of 0.70, as shown in Table 3. The confusion matrix shows high accuracy for predicting minimum tillage (47.47%), moderate accuracy for no till (24.29%), and lower accuracy for conventional till (11.62%) (Supplementary Table 5). Overall, the model exhibited a greater recall rate of 90% for minimum tillage compared to 71% for conventional tillage.

The distribution of tillage practices varied across eastern SD (Fig. 5). In 2022 and 2023, no-tillage techniques were used on 43.7 and 53.7% of the total land planted with corn and soybeans. In 2023, 15.8% of the fields were planted using conventional tillage methods. In 2022 and 2023, more than 82% of the fields previously planted with corn and 70% of the fields previously planted with soybeans were managed with conservation tillage (minimum or no-tillage) practices (Table 4). In 2022 and 2023, no-tillage practices were widely used in the western portions of eastern SD, mostly in the counties surrounding the Missouri River. In contrast, the counties of Moody, Roberts, Marshall, and Brookings in the eastern section of the study area primarily employed conventional tillage methods. We also found that the counties of Turner, Lincoln, Lake, and Moody were the main regions where minimum tillage was particularly prominent.

**Table 4:** Distribution of tillage practices based on the previous year’s crops for 2022 and 2023

Year	Type of tillage	Previous crop				Total area	
		Corn		Soybean			
2022	Conventional Tillage	27.4%	1,827 km^2^	35.6%	2,373 km^2^	16.5%	6,668 km^2^
	Minimum tillage	23.7%	2,864 km^2^	27.1%	3,275 km^2^	29.8%	12,084 km^2^
	No-tillage	48.7%	10,590 km^2^	37.1%	8,061 km^2^	53.7%	21,745 km^2^
2023	Conventional Tillage	14.4%	927 km^2^	26.9%	1,732 km^2^	15.8%	6,439 km^2^
	Minimum tillage	32.5%	5,307 km^2^	43.5%	7,103 km^2^	40.3%	16,330 km^2^
	No-tillage	53.0%	9,396 km^2^	29.4%	5,212 km^2^	43.7%	17,728 km^2^

PPT_T_ (Precipitation), NDI5, wind_T_ (wind speed), SSM_T_ (soil moisture), and albedo, which serve as proxies for land surface properties related to climate, moisture, and green cover change, were shown to be the more relevant factors influencing the identification of tillage classes. Soils with clay concentrations above 25% were more likely to be conventional tilled than soils with less than 10% clay, particularly in the eastern part of the study area (e.g., Deuel, Brookings, Hamlin counties).

We observed a greater probability of conventional tillage in Roberts and Marshall counties, which experienced higher precipitation and surface soil moisture in fall and spring (Supplementary Figs. 4 and 5). Conventional and minimum tillage were most commonly used in counties with higher land surface temperatures, such as Clay and Yankton counties. Incorporating short-wave infrared bands, specifically B11, into the model helped differentiate among the tillage practices. The order of the tillage indices from highest to lowest considering the CatBoost feature important scores was NDTI, NDRI, CRC, and NDI5.

The study revealed that a substantial portion of the cover cropped areas, approximately 45% (882 km^2^) in 2022 and 52% (1029 km^2^) in 2023, were also managed without tillage. Notably, only 6% of the cover cropped fields utilized conventional tillage in the spring of 2023 following the termination of cover crops. Our results demonstrate a positive correlation between the use of cover crops and conservation tillage practices.

## Discussion

### Cover Crop Distribution

Our study evaluated the capability of visible, near infrared (NIR), and shortwave-infrared (SWIR) indices, along with environmental variables, to predict cover crop presence in eastern SD. Overall, we found that cover crops were particularly prevalent in the southern (e.g., Bon Homme and Yankton counties) and eastern regions (e.g., Deuel and Codington counties) of eastern SD, which experience higher temperatures and more precipitation). For example, southern counties in eastern South Dakota accumulated more growing degree days (GDDs) compared to northern counties in the study area. This regional variation aligns with findings from studies conducted by Kc et al. (2021) and Seifert et al. (2018) in the U.S. Midwest emphasizing, that cover crops planted early in the fall have a higher likelihood of survival. These crops require sufficient GDDs to establish before winter, highlighting the importance of environmental conditions in their successful implementation(Chatterjee et al. 2020).

We found that NDVI, a measure of greenness, is the most important predictor of cover crop presence and absence. Aside from agricultural weeds and perennial features such as grassed waterways, cover crops are the primary green cover expected within corn and soybean fields in the late fall. We also found that NDTI, which utilizes SWIR bands, was a significant predictor in our cover crop model. The use of satellite imagery from November, which typically falls within the post-harvest period, underscores the significance of NDTI in detecting cover crops. During this period, fields either undergo tilling, or retain a substantial amount of crop residue. The NDTI helps differentiate these scenarios: fields with cover crops exhibit different spectral characteristics compared to tilled fields, which are often bare or have minimal residue. Thus, the NDTI is effective in identifying the presence or absence of cover crops by distinguishing them from tilled fields.

We found that the vast majority (~80%) of cover crops were planted after the harvest of corn rather than soybeans. Our model did not, however, differentiate between corn harvested for grain and corn harvested for silage. Previous research and local expertise indicate that farmers are more likely to plant cover crops in this region following corn harvested for silage, which typically occurs by mid-September, than corn harvested for grain (Hively et al. 2015). The combination of harvesting corn for silage followed by the planting of a cover crop is more likely for two primary reasons. First, the cover crop can be planted sooner in the fall, which allows the cover crop more time to grow in the fall (Nowak et al. 2021; Seifert et al. 2018). Second, farmers who harvest corn for silage are generally near livestock, and the cover crop biomass can serve as livestock feed. For example, Bon Homme County, which contains the most extensive Hutterite colony in North America (Riley and Johnson 1970), had more than six times the use of cover crops than the rest of eastern SD. Farms within the Hutterite colony are known to use integrated crop-livestock practices (Riley and Johnson 1970).

### Tillage Practices Distribution

We used Sentinel-2 bands, tillage indices, textural features, and environmental variables to identify tillage practices in the study area. Our findings highlight the significant influence of pedoclimatic conditions on the adoption of different tillage methods. Our observations indicate that conventional tillage was the prevailing method in the eastern sections. In contrast, no tillage is the predominant practice as we move towards the western regions of eastern SD. These geographical variations in tillage practices might be due to climatic shifts from east to west and precipitation-soil moisture variability. No-tillage systems have shown better soil water-use efficiency than conventional plow tillage (Wang et al. 2020). In the eastern part of the study area, where the soil is moist and favorable for tile drainage, it may be necessary to till the soil to incorporate residues and facilitate decomposition. In contrast, in the semiarid western regions of eastern SD, where precipitation is less abundant, the absence of tilling can conserve soil moisture by maintaining crop residue coverage and decreasing soil water evaporation (Blevins et al. 1971; Wang et al. 2020).

Land surface temperature emerged as the primary indicator in our tillage model. LST measures the emission of thermal radiance from the surface of the canopy in vegetated areas or bare land, where incoming solar energy interacts with and increases ground temperature (Khan et al. 2021). Counties in the northeastern part of the study area, such as Roberts, Day, and Grant, have lower LSTs compared to counties in the southern region (Supplementary Figs. 4 and 5). This decrease in LST reduces the rate at which soil warms, potentially impacting plant growth dynamics. Consequently, these regions may experience increased adoption of conventional tillage practices. Conventional tillage involves incorporating crop residues into the soil, which enhances the absorption of incoming solar radiation and consequently raises soil temperatures. In contrast, conservation tillage practices leave crop residues on the soil surface, mitigating soil temperature increases (Brien and Daigh 2019; Turmel et al. 2015). Studies by Shen et al. (2018) and Barnes et al. (2021) have also emphasized the importance of surface thermal data in identifying areas with varying levels of crop residue coverage and tillage.

We observed that the soils in counties such as Codington, Clay, Day and Marshall have high clay content (25–40%) and silt content (58–72%) with poor internal drainage (Westin 1951) (Supplementary Fig. 3). In these areas, tillage and sowing operations typically occur following fall precipitation when the soil is damp and prone to compaction (Chan et al. 2006). This compaction reduces the availability of moisture and nutrients for root uptake, ultimately leading to lower crop yields (Morrison et al. 2017). Consequently, conventional tillage methods are commonly employed to alleviate compaction and improve soil aeration and infiltration (Bilen et al. 2010). Conversely, in regions with sandy loam soils, which are prevalent in western parts of the study area, rapid drainage and erosion susceptibility encourage farmers to forego tillage (Bilen et al. 2010; Westin 1951). These soils benefit from reduced disturbance, which helps maintain soil structure and moisture levels critical for crop productivity.

The variable importance of the CatBoost model showed that SWIR-based indices and bands enhanced the prediction capacity for detecting tillage practices. The results also showed that NDTI and B11 had higher relative importance than the other visible and NIR-based variables. These results indicate that crop residues and soil exhibit similar spectral characteristics in the visible and near-infrared regions but differ significantly in the short-wave infrared region. Therefore, the most effective way to distinguish between crop residues and soil is to analyze the spectral absorption characteristics of lignin and cellulose in crop residues, particularly around the 2100 nm wavelength (Hively et al. 2018; Nagler et al. 2003). Furthermore, B8 and NDVI were among the top predictors for detecting conservation tillage. However, their contribution lagged behind SWIR-based indices, as they cannot distinguish between crop residues and soils. This limitation arises because NIR-based variables like NDVI cannot effectively discriminate between crop residues and soils due to their similar reflectance properties in these spectral regions (Cai et al. 2019; Hively et al. 2018; Sullivan et al. 2008).

Furthermore, our results found that a notable proportion of farmers implemented both conservation tillage and cover cropping, reflecting a growing trend towards implementing conservation agriculture. Although only 4% of the fields had cover crops, this overlap indicates that these practices can be effectively combined. It is likely that farmers who adopt cover crops are already practicing conservation tillage.

Our analysis revealed a low recall of 0.71 in conventional tillage fields, likely influenced by varying amounts of crop residue left behind by corn or soybeans in different fields. This residue from the previous crops significantly affected the classification outcome, introducing ambiguity into the model’s analysis. Moreover, our findings indicate that GLCM textural features did not serve as effective variables for tillage mapping. GLCM texture metrics are sensitive to spatial patterns in image data, but they may not adequately capture the diverse residue patterns and soil conditions resulting from different tillage practices (e.g., disking, moldboard plowing, chisel plowing).These different practices create distinct residue patterns and soil dispersal, making it challenging to combine them into a single category at a 10-meter resolution (Najafi et al. 2018a; Xiang et al. 2022). The performance of Sentinel-1 data in our study was also compromised due to operational issues with Sentinel-1B. The failure of Sentinel-1B in December 2021 resulted in a lack of data availability in our study region during critical periods.

### Validations

The cover crop and tillage models demonstrated robust accuracy, exceeding 80%, and reasonable kappa values. A comparison of the estimated maps with the Natural Resources Conservation Service (NRCS) 2022 county-level cover crop and tillage estimates helped validate our findings. The NRCS 2022 county-level cover crop estimates showed that cover crops were more common in the western and central parts of the eastern SD. This matches our estimates of counties where a lot of land is covered by cover crops (USDA-NASS, 2024). The 2022 NRCS estimates showed that the eastern regions dominated conventional tillage, while the western part of the state had a higher prevalence of no tillage (USDA-NASS 2022).

The Conservation Technology Operations Center published another dataset on tillage and cover crops (Gustafson et al. 2019), wherein most of the conservation tillage acres were concentrated in the Northwestern (Hyde, Sully, Hand) regions of eastern SD. Conversely, their latest published map of 2021 indicated a high density of cover crops in Brookings, Minnehaha, and Codington counties, which aligns with our model findings. Seifert et al. (2018) used Landsat images to derive cover crop maps for the US Midwest from 2008 to 2016, and their results align with our findings that a comparatively lower portion (<10%) of the eastern SD is managed with cover crops. Furthermore, the findings of Azzari et al. (2019) align with our study, as the county-level estimates of tillage maps from 2008 to 2016 indicate a continuous pattern of declining tilling intensity towards the west and a prevalence of high-intensity tillage in the eastern regions of the state.

### Limitations and Future Scope

Although our study significantly enhanced the capacity to detect the presence of cover crops and tillage on a wide spatial scale, it has certain noteworthy shortcomings that could be addressed by further research. The field calibration dataset used to derive the models was limited in spatial extent to a few counties in the study regions. We also observed a higher recall rate in no-tillage fields than in conventional tillage fields. One potential factor contributing to this disparity could be that low-intensity tillage practices tend to leave a greater amount of crop residue cover in corn fields than in soybean-cultivated fields. Consequently, incorporating historical data related to previous cash crops should be viewed as a crucial component to be considered in future research initiatives aimed at improving the model’s predictive capabilities. Moreover, it is important to recognize that certain areas marked as having been planted with cover crops may not display sufficient vegetative growth to be detected by the satellite images used in this study for estimating ground coverage. Conversely, regions with significant weed infestations may be classified as covered. Additionally, incorporating data on corn silage and mapping the geographic distribution of dairy farms could improve the predictive capabilities of our model while investigating the influence of farm structure on the adoption of cover cropping.

Improved spaceborne sensors offer data beyond greenness for cover crop and tillage recognition. Researchers can study cover crop species and identify plants from crop residues and barren soils using hyperspectral imagery. The increasing accessibility of thermal data with enhanced spatial resolution, shown by the Ecosystem Spaceborne Thermal Radiometer Experiment on Space Station (Hook and Fisher 2019), allows additional research into the potential of thermal data for predicting agricultural land-use practices.

It is important to gain a more comprehensive understanding of farmers’ subjective beliefs, including their opinions regarding the significance of soil health and the economic advantages associated with cover cropping and no tillage. This understanding may offer valuable insights for future research and extension endeavors to facilitate informed adoption decisions. Furthermore, farmers’ participation in cost-sharing programs and knowledge of CA benefits are necessary to encourage the adoption of cover crops. We acknowledge that sustained efforts to enhance precision are imperative, considering the potential future utility of remote sensing for policy monitoring and contributions.

## Conclusions

Our results suggest that satellite-derived cover crop and tillage maps can be rapidly produced in the eastern SD with high confidence. Adopting conservation agriculture, such as conservation tillage and cover crops, can help diversify farm-level risks and promote on-farm biodiversity in the US Corn Belt. In this context, it is important to design appropriate strategies to increase the adoption of conservation practices and promote regenerative agriculture that protects and restores ecosystem services. We predicted the spatial distribution of cover crops and tillage practices across eastern SD using the CatBoost classifier trained with field samples and environmental drivers from different remote sensing datasets. This paper presents an analysis of the regional and temporal patterns of these activities and examines the environmental factors that influence farmers’ decision-making process. Specifically, our findings indicate that factors such as geographical location, climate, and soil patterns of a farm can significantly impact the likelihood of adopting conservation agriculture practices in the future. The outcomes of this study may offer valuable insights into potential areas for future research and extension priorities. Our study contributes to the development of a measurement, reporting, and verification (MRV) solution that can be utilized to monitor various climate-smart practices in agriculture. This approach can benefit both public and private sector organizations by enabling them to track landscapes remotely and efficiently. This, in turn, can incentivize farmers to adopt conservation practices, contributing to climate change mitigation efforts and fostering sustainable agricultural development.

## Supplementary information

Supplementary Information

## Data Availability

Field data were collected by a commercial company [Agreena] and cannot be shared. Derived data/maps supporting the findings of this study are available from the corresponding author on request. The cover crops and tillage layers can be viewed on the GEE app using the links “Cover crop” and “Tillage”.
