# Supplementary material for: Monitoring the Spatial Distribution of Cover Crops and Tillage Practices Using Machine Learning and Environmental Drivers across Eastern South Dakota
Source: Environ Manage. 2024 Jul 29;74(4):742–56. doi: 10.1007/s00267-024-02021-0 (PMC11392983; doi:10.1007/s00267-024-02021-0)
Supplement: Supplementary file 1 — Supplementary Information [file 267_2024_2021_MOESM1_ESM.docx]

**Monitoring the spatial distribution of cover crops and tillage practices using machine learning and environmental drivers across eastern South Dakota.**

Khushboo jain^1*^, Ranjeet John^1, 2^, Nathan Torbick^3^, Venkatesh Kolluru^1^, Sakshi Saraf^2^, Abhinav Chandel^1^, Geoffrey M. Henebry^4,5^, Meghann Jarchow^1^

*Corresponding author: Khushboo jain; Email: [khushboo.jain@usd.edu](mailto:khushboo.jain@usd.edu); ORCID ID: 0009-0001-9843-1495

1. Department of Sustainability and Environment, University of South Dakota, Vermillion, SD 57069, USA

2. Department of Biology, University of South Dakota, Vermillion, SD 57069, USA

3. Agreena, Langebrogade 3F, 3rd Floor, 1411 Copenhagen K,Denmark

4. Department of Geography, Environment, and Spatial Sciences, Michigan State University, East Lansing, MI 48823, USA

5. Center for Global Change and Earth Observations, Michigan State University, East Lansing, MI 48823, USA

Table 1 Sentinel 2A (10m) derived vegetation and tillage indices used in this study (abbreviations in S.I Table 3).

| VI | Formula | Notes | Reference |
| --- | --- | --- | --- |
| NDVI | NDVI$= \frac{\rho_{\mathrm{nir}} -\rho_{\mathrm{red}}}{\rho_{\mathrm{nir}}+\rho_{\mathrm{red}}}$ | $\rho_{\mathrm{nir}}$and $\rho_{\mathrm{red}}$ are Sentinel 2A band 8 and 4 | (Rousel et al., 1973) |
| NDTI | NDTI = $\frac{\rho_{swir1}-\rho_{swir2}}{\rho swir1+\rho_{swir2}}$ | $\rho_{swir1}$and $\rho_{swir2}$ are Sentinel 2A band 11 and 12 | (Van Deventer et al., 1997) |
| NDI5 | NDI5 = $\frac{\rho_{\mathrm{nir}}-\rho_{swir1}}{\rho_{\mathrm{nir}}+\rho_{swir1}}$ | $\rho_{\mathrm{nir}}$and $\rho_{swir1}$are Sentinel 2A band 8 and 11 | (McNairn & Protz, 1993) |
| NDI7 | NDI7 = $\frac{\rho_{\mathrm{nir}}-\rho_{swir2}}{\rho_{\mathrm{nir}}+\rho_{swir2}}$ | $\rho_{\mathrm{nir}}$ and $\rho_{swir2}$ are Sentinel 2A band 8 and 12 | (McNairn & Protz, 1993) |
| NDRI | NDRI = $\frac{\rho_{swir2}-\rho_{\mathrm{red}}}{\rho_{swir2}+\rho_{\mathrm{red}}}$ | $\rho_{swir2}$ and $\rho_{\mathrm{red}}$ are Sentinel 2A band 12 and 4 | (Gelder et al., 2009) |
| SNDVI | SNDVI = $\frac{\rho_{\mathrm{nir}}-\rho_{\mathrm{red}}}{(\rho_{\mathrm{nir}}+\rho_{\mathrm{red}}+0.16)}$ | $\rho_{\mathrm{nir}}$and $\rho_{\mathrm{red}}$ are Sentinel 2A band 8 and 4 | (Jiang et al., 2006) |
| CRCI | CRCI = $\frac{\rho_{swir1}-\rho_{\mathrm{green}}}{\rho_{swir1}+\rho_{\mathrm{green}}}$ | $\rho_{swir1}$and $\rho_{\mathrm{green}}$ are Sentinel 2A band 11 and 3 | (Sullivan et al., 2008) |

Table 2 Gray level co-occurrence matrix (GLCM) textural features

| Index | Formula | Description |
| --- | --- | --- |
| Contrast | $\sum_{I,J=0}^{N-1} P_{i,j}{(i-j)}^{2}$ | A measure of local variation in pixel values among neighboring pixels |
| Correlation | $\sum_{I,J=0}^{N-1} P_{i,j}\left[ \frac{\left( i-u_{i} \right)(j-\mu_{i})}{\sqrt{{(\sigma}_{i}^{2})(}{(\sigma}_{j}^{2})} \right]$ | Linear dependency of pixel values on those of neighboring pixels |
| Entropy | $\sum_{I,J=0}^{N-1} P_{i,j}(-1n P_{i,j})$ | High when the pixel values of the GLCM have varying values. |
| Variance | $\sigma^{2}=\sum_{I,J=0}^{j_{\max}} \{\left( i-\mu\right)^{2}h_{i}\}$ | Measures the dispersion of the gray-level distribution to emphasize the visual edges of land-cover patches |
| Angular surface moment | $\sum_{ij=0}^{n-1} {P_{\mathrm{ij}}}^{2}$ | Measure of the order in an image |
| Shade | $\sum_{i=0}^{n-1} \sum_{j=0}^{n-1} {\{i-j)}^{2}{P_{(i,j)}}$ | The lack of proper symmetry in an image |
| Mean/Average | $\mu_{i}=\sum_{I,J=0}^{N-1} i_{( P_{i,j})}$ | Measures the mean of the gray level sum distribution of the image |
| Homogeneity | $\sum_{i} \sum_{j} \frac{1}{1+{(i-j)}^{2}} p^{(i,j)}$ | Represents the homogeneity of the gray level distribution within the image objects |
| Inertia | $\sum_{i=0}^{n-1} \sum_{j=0}^{n-1} \left\{ i+j-u_{x-}u_{y} \right\}^{4}P_{(i,j)}$ | The similarity in gray levels between neighboring pixels |

Table 3 Description and source details of the variables considered for the study.

| Variables | Codes and units | Spatial Resolution | Description |
| --- | --- | --- | --- |
| Climatic |  |  |  |
| Mean vapor pressure deficit | VPD [kPa] | 4.6 km | Gridded Surface Meteorological dataset |
| Wind velocity | Wind_speed [m/s] | 10 m | Gridded Surface Meteorological dataset |
| Land Surface Temperature | Lst [K] | 1 km | MODIS MOD11A1 V6.1 derived land surface temperature (LST) and emissivity values |
| Albedo | Albedo | 500 m | MODIS MCD43A3 V6.1 Albedo daily 16-day product |
| Growing degree days | tmean | 4.6 km | PRISM Daily mean temperature (calculated as (tmin+tmax)/2) |
| Daily total precipitation | Ppt [mm] | 4.6 km | Daily total precipitation (including rain and melted snow) |
| Biophysical  Soil Moisture | Ssm[km] | 10 km | NASA-USDA Enhanced SMAP |
| Surface texture | Surf_text | 10 m | Gridded Soil Survey Geographic (gSSURGO) derived surface texture |
| Slope | Slope [degrees] | 30m | NASA- Shuttle Radar Topography Mission (SRTM) |
| Normalized Difference Vegetation Index | NDVI | 10m | Seasonal median composites for each surface reflectance was derived from Sentinel 2A |
| Normalized Difference Tillage Index | NDTI | 10m |  |
| NDI5 | NDI5 | 10m |  |
| NDI7 | NDI7 | 10m |  |
| Senescent Normalized Difference Vegetation Index | SNDVI | 10m |  |
| Normalized Difference Residue Index | NDRI | 10m |  |
| Sentinel 2A-Blue | B2 | 10m |  |
| Sentinel 2A-Green | B3 | 10m |  |
| Sentinel 2A-Red | B4 | 10m |  |
| Sentinel 2A-Near InfraRed | B8 | 10m |  |
| Sentinel 2A-Short Wave InfraRed 1 | B11 | 20m |  |
| Sentinel 2A-Short Wave InfraRed 2 | B12 | 20m |  |
| Sentinel-1 SAR | VV | 10 m | Seasonal median composites derived from Sentinel-1 dual-polarization C-band |
|  | VH | 10 m |  |

Table 4 Confusion Matrix of cover crops classification model

|  | | Predicted Class (%) | | |
| --- | --- | --- | --- | --- |
|  |  | Presence | Absence | Total |
| Actual Class (%) | Presence | 40 | 5.19 | 45.19 |
|  | Absence | 9.63 | 45.19 | 54.82 |
|  | Total | 49.63 | 50.38 | 100 |

Table 5 Confusion Matrix of tillage types classification model

|  | | Predicted Class (%) | | | |
| --- | --- | --- | --- | --- | --- |
|  |  | Conventional till | Minimum till | No till | Total |
| Actual Class (%) | Conventional till | 11.62 | 4.02 | 0.78 | 16.42 |
|  | Minimum till | 2.51 | 47.47 | 2.55 | 52.53 |
|  | No till | 0.75 | 6.01 | 24.29 | 31.05 |
|  | Total (%) | 14.88 | 57.5% | 27.62 | 100 |


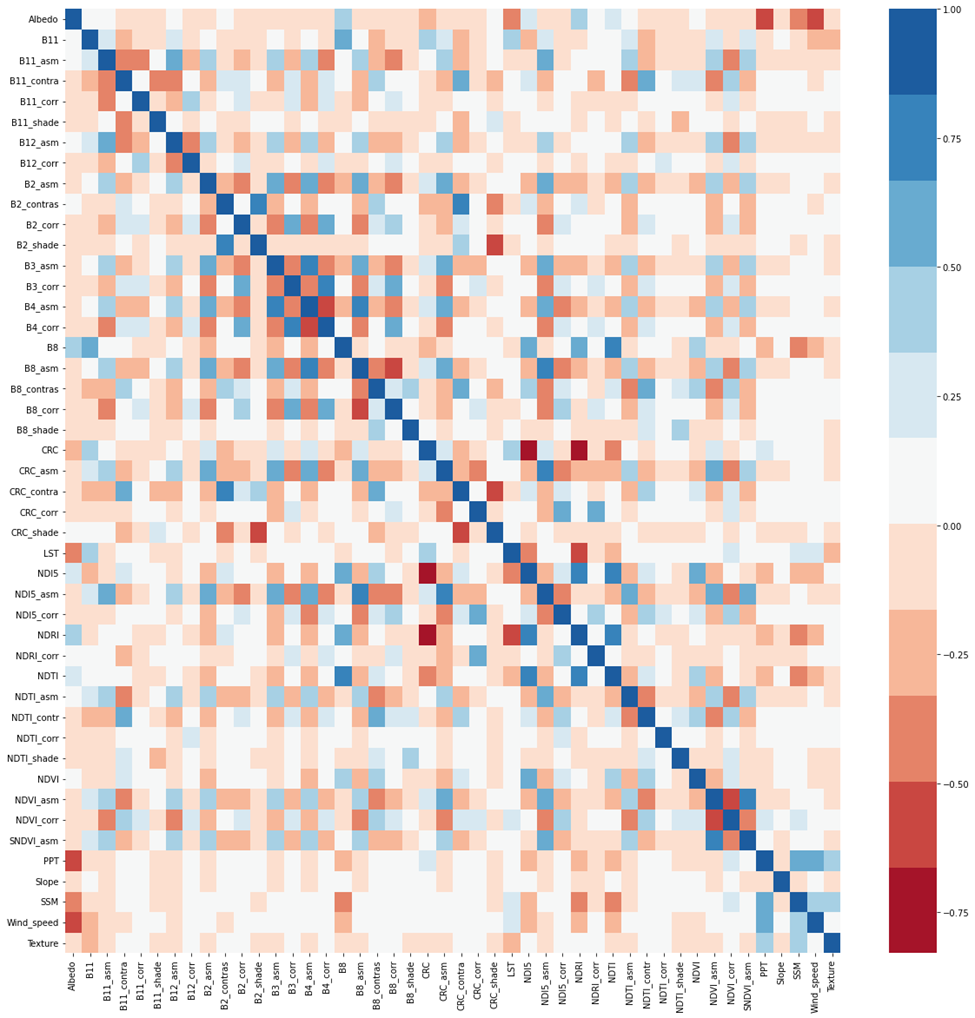


Fig.1 Correlation plot for tillage variables (R<0.8)


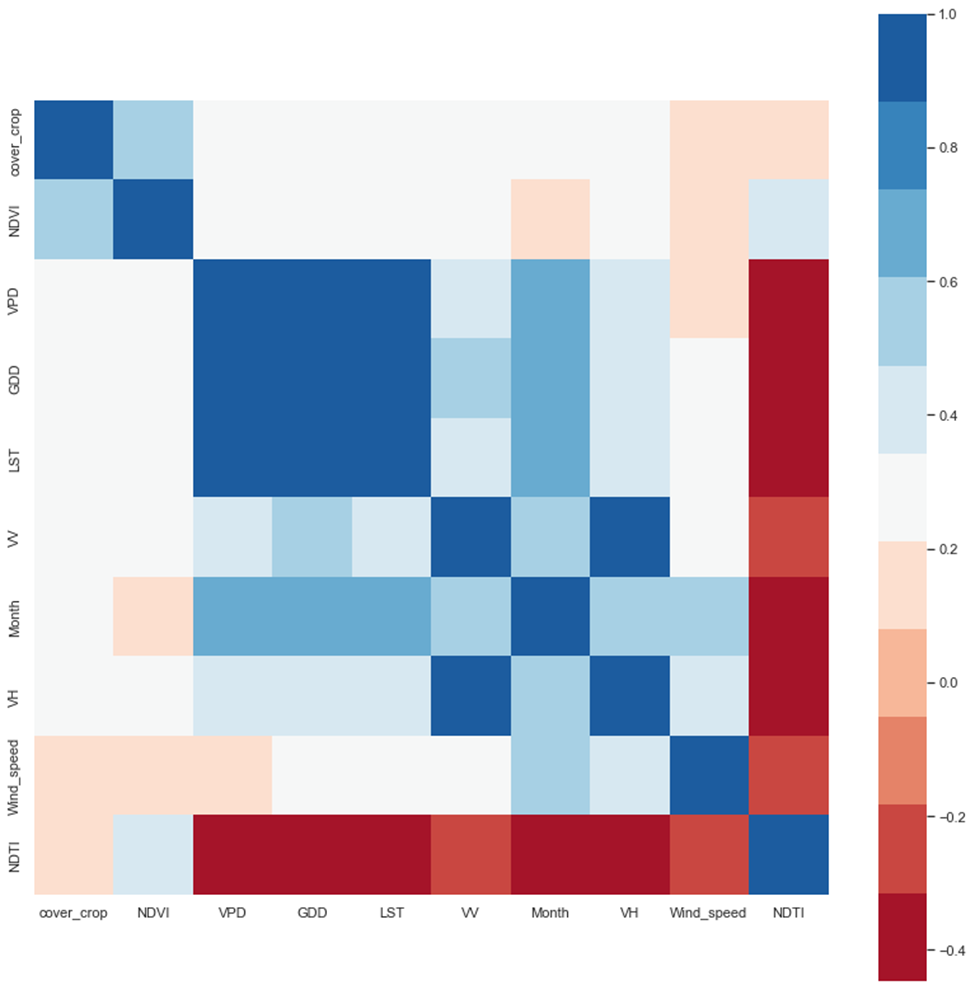


Fig. 2 Correlation plot for cover crop variables (R<0.8)


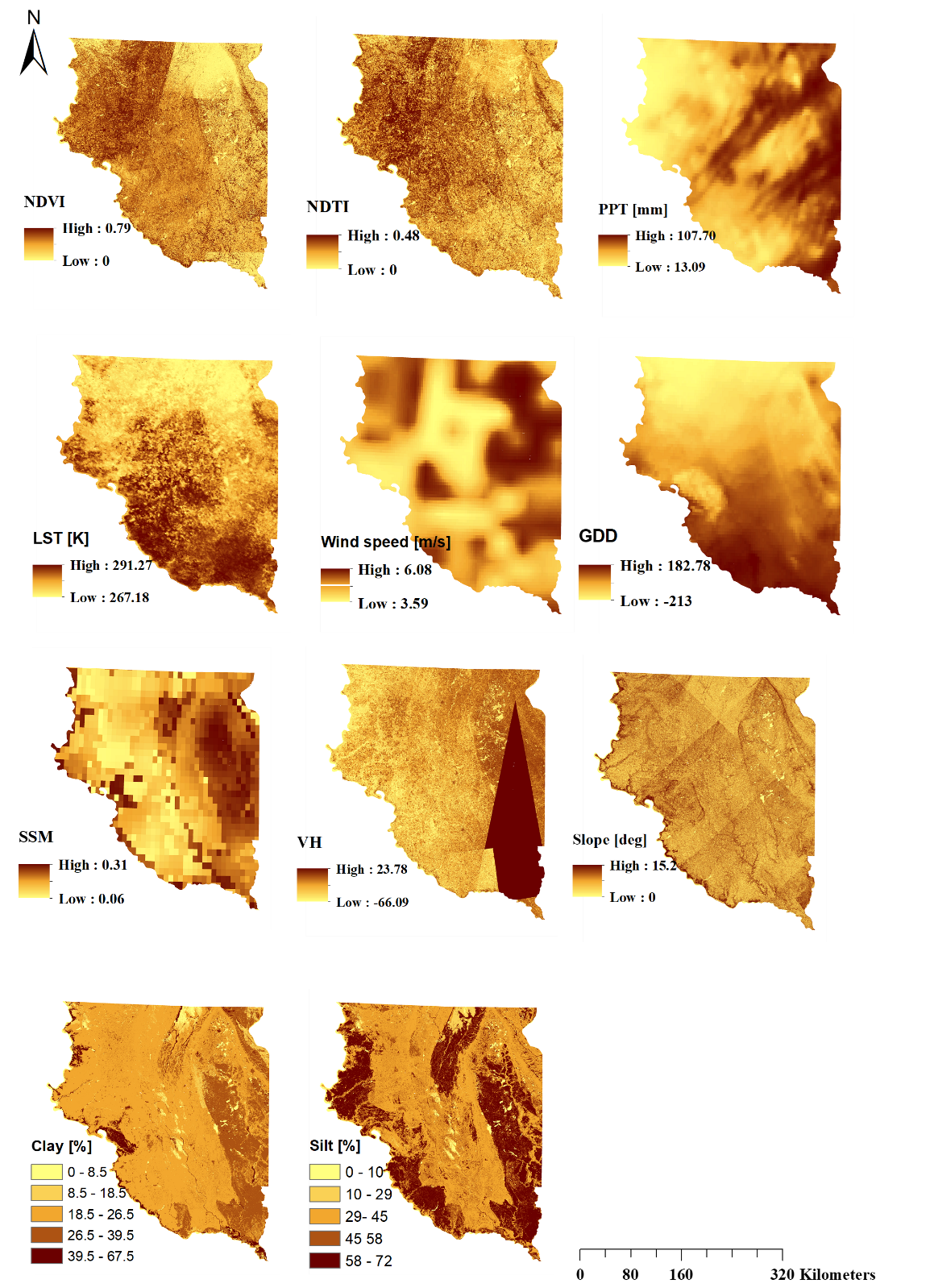


Fig. 3 Climate and Sentinel derived variables used in this study for predicting tillage and cover crops distribution (Fall 2022) (a) Normalized difference vegetation index (NDVI) (b) Normalized difference tillage index (NDTI) (c) fall precipitation (d) fall land surface temperature (e) fall wind speed (f) Growing degree days (GDD) (g) fall surface soil moisture (h) Sentinel-1 VH band (i) slope (degree) (j) clay (%) (k) silt


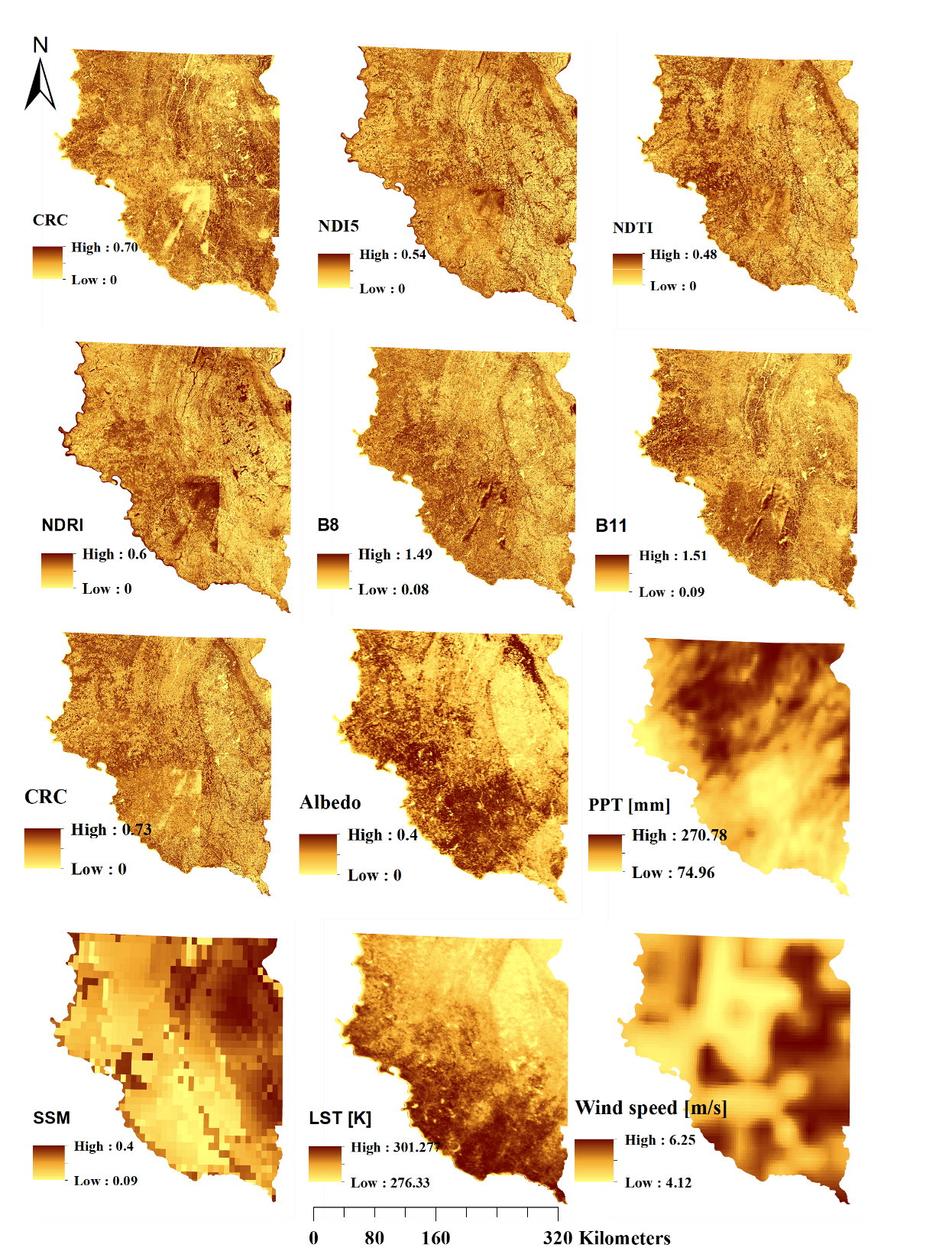


Fig. 4 Climate and Sentinel derived variables used in this study for predicting tillage (spring 2022) (a) Crop residue cover index (CRCI) (b) Normalized difference Index 5 (c) Normalized difference tillage index (d) Normalized Difference Residue Index (e) Sentinel Band-8 (f) Sentinel Band-11 (g) Normalized difference vegetation index (NDVI) (h) Albedo (i) spring precipitation (j) spring surface soil moisture (k) Spring land surface temperature (l) spring wind speed


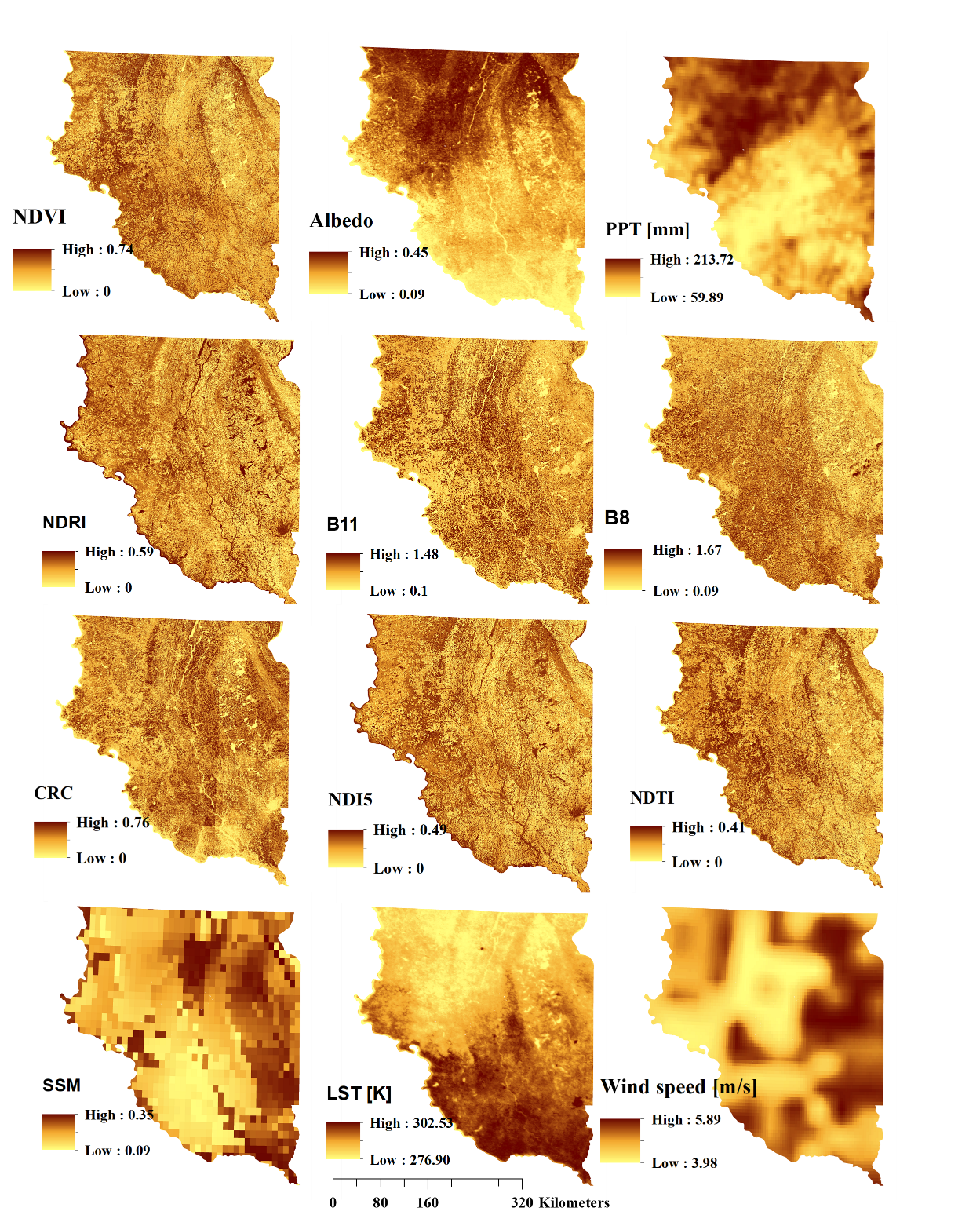


Fig. 5 Climate and Sentinel derived variables used in this study for predicting tillage (spring 2023) (a) Normalized difference vegetation index (NDVI) (b) Albedo (c) spring precipitation (d) Normalized Difference Residue Index (e) Sentinel Band-11 (f) Sentinel Band-8 (g) Crop residue cover index (CRCI) (h) Normalized difference Index 5 (i) Normalized difference tillage index (NDTI) (j) spring surface soil moisture (k) Spring land surface temperature (l) spring wind speed


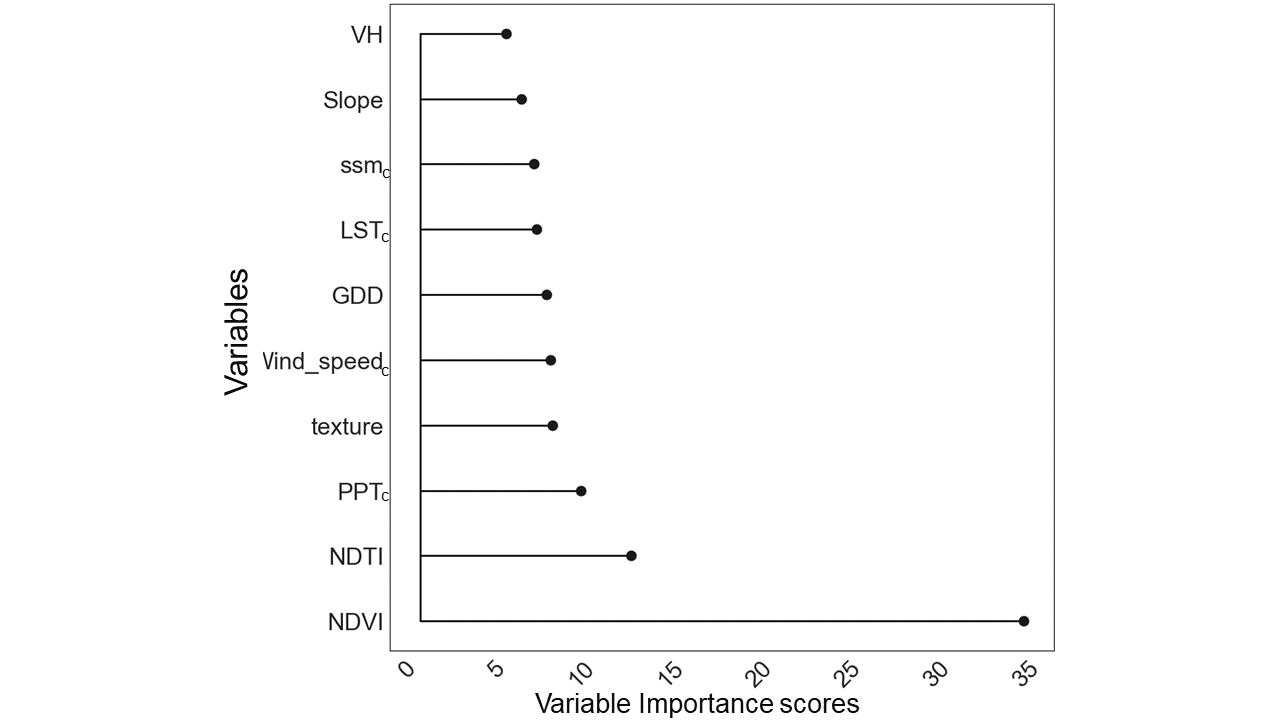


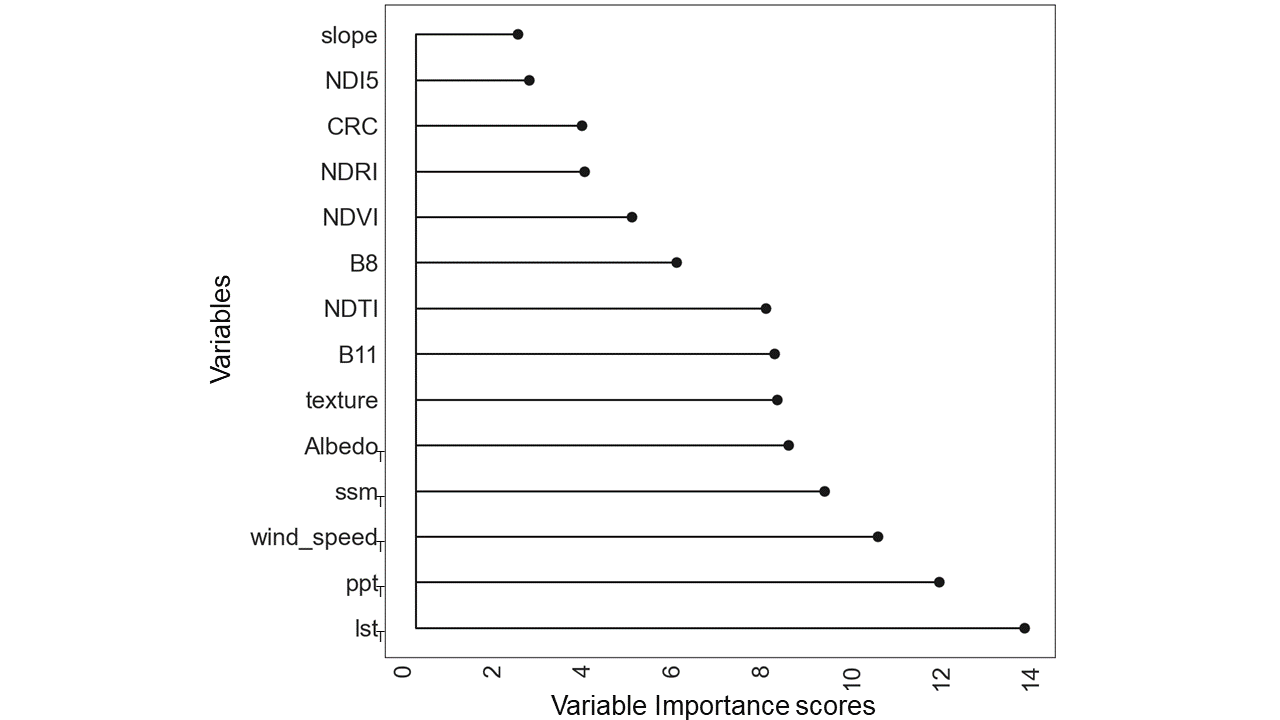


*Note- Refer Table 2 for the acronyms of the variables listed in the graphs.

Fig.6. and Fig.7. Variable importance of (a) cover crops and (b) tillage type classification model.
